# Whole genome sequencing enables the characterization of BurI, a LuxI homologue *of Burkholderia cepacia* strain GG4

**DOI:** 10.7717/peerj.1117

**Published:** 2015-08-06

**Authors:** Kah Yan How, Kar Wai Hong, Kok-Gan Chan

**Affiliations:** Division of Genetics and Molecular Biology, Institute of Biological Sciences, Faculty of Science, University of Malaya, Kuala Lumpur, Malaysia

**Keywords:** *Burkholderia cepacia*, Quorum sensing, AHL synthase, Protein expression, Liquid chromatograhy mass spectrometry (LC-MS), *N*-acylhomoserine lactone (AHL)

## Abstract

Quorum sensing is a mechanism for regulating proteobacterial gene expression in response to changes in cell population. In proteobacteria, *N*-acyl homoserine lactone (AHL) appears to be the most widely used signalling molecules in mediating, among others, the production of extracellular virulence factors for survival. In this work, the genome of *B. cepacia* strain GG4, a plasmid-free strain capable of AHL synthesis was explored. *In silico* analysis of the 6.6 Mb complete genome revealed the presence of a LuxI homologue which correspond to Type I quorum sensing. Here, we report the molecular cloning and characterization of this LuxI homologue, designated as BurI. This 609 bp gene was cloned and overexpressed in *Escherichia coli* BL21(DE3). The purified protein was approximately 25 kDa and is highly similar to several autoinducer proteins of the LuxI family among *Burkholderia* species. To verify the AHL synthesis activity of this protein, high resolution liquid chromatography-mass spectrometry analysis revealed the production of 3-oxo-hexanoylhomoserine lactone, *N*-octanoylhomoserine lactone and 3-hydroxy-octanoylhomoserine lactone from induced *E. coli* BL21 harboring the recombinant BurI. Our data show, for the first time, the cloning and characterization of the LuxI homologue from *B. cepacia* strain GG4 and confirmation of its AHL synthesis activity.

## Introduction

It has been widely accepted that single-celled bacteria communicate with each other using small, hormone-like chemical molecules known as autoinducers. Such cell–cell communication mechanism or quorum sensing (QS) regulates various physiological activities among bacterial communities, ranging from bioluminescence to swarming motility (Dong & Zhang, 2005; Miller & Bassler, 2001; Williams, 2007). The QS bacteria release autoinducers in response to environmental stimuli or at specific stages of growth. At a threshold level, these signalling molecules will then bind to their cognate receptor to form an autoinducer-receptor complex that regulate the expression of target genes (Chan et al., 2011b; Hong et al., 2012b; Williams et al., 2007). By far, the most extensively studied QS molecules in the last two decades is *N*-acyl homoserine lactone (AHL) (Eberhard et al., 1981). Other well-known bacterial cell–cell communication signals include cyclic thiolactone (Ji, Beavis & Novick, 1995), hydroxyl-palmitic acid methyl ester (PAME) (Flavier et al., 1997), furanosylborate (Chen et al., 2002), and methyl dodecenoic acid (Wang et al., 2004).

Among Gram-negative bacteria, a myriad of AHL derivatives which differ in length or structure of the acyl side chain, have been identified. The acyl side chain consists of fatty acids which have different chain length, degree of saturation, and the presence of substituent at the C3 position (Swift et al., 1997). The two principal components of AHL-driven QS systems are LuxI and LuxR proteins which act as the AHL synthase and signal receptor, respectively (Fuqua, Parsek & Greenberg, 2001). The secreted AHL will bind to LuxR protein to form LuxR/AHL complex which then regulates the expression of target genes and thus the physiological functions of the cell (Chong et al., 2012; Parsek & Greenberg, 2000). Studies have shown that the *luxI* gene is one of the main targets of the LuxR/AHL complex, thus increasing the production of AHL (Hong et al., 2012b). LuxI/LuxR QS systems have been well studied in numerous bacterial species. In addition, whole genome sequencing projects have unravelled more bacterial species with putative *luxI/luxR* homologues. There were also multiple systems of QS found in single genomes (Hao et al., 2010).

In the past few decades, members of the *Burkholderia* genus are among groups of Proteobacteria which have been extensively studied in QS system. These Gram-negative bacteria are versatile microorganisms and may cause a number of diseases in many host organisms. They have been isolated from water, soil, industrial areas and hospital environments (Stoyanova et al., 2007). In recent years, the genus *Burkholderia* has been phylogenetically well defined. It comprises more than 60 species which are functionally remarkably diverse. Of all *Burkholderia* species, *B. cepacia* is of greatest importance. Previously known as a phytopathogen and the etiological agent of soft rot of onions, *B. cepacia* (previously *Pseudomonas cepacia*) is also an important causative agent in patients with cystic fibrosis (Govan, Hughes & Vandamme, 1996). QS in *B. cepacia* was found to play critical roles in regulation and expression of extracellular proteins and regulation of swarming and biofilm formation (Aguilar et al., 2003).

Chan and his co-workers have been exploring novel rhizosphere environments for bacterial communities in the Malaysian rainforest and recently, and the genus *Burkholderia* was recently found associated with the roots of *Zingiber officinale *(ginger) (Chan et al., 2011a; Chan et al., 2011b). One of the ginger rhizosphere strains was identified as *B. cepacia* strain GG4 (hereafter referred to as strain GG4). This soil isolate was found to secrete four AHLs, namely 3-oxo-hexanoyl-homoserine lactone (3-oxo-C6-HSL), *N*-octanoyl-L-homoserine lactone (C8-HSL), 3-hydroxy-octanoyl-homoserine lactone (3-hydroxy-C8-HSL) and *N*-nonanoyl-L-homoserine lactone (C9-HSL). While most *Burkholderia* spp. have been reported to produce C6-HSL, C8-HSL and 3-hydroxy-C8-HSL, strain GG4 was the first *Burkholderia* strain found to synthesize long-chain C9-HSL. The production of C9-HSL may regulate unknown genetic traits which could play a vital role in the adaptation of this strain GG4 as endophytic bacterium in ginger rhizosphere, as compared to other *Burkholderia* species. Hence, it is of high interest to elucidate the role of the AHLs as the global regulator of QS activity in physiological functions of this soil-dwelling bacterium.

The whole-genome sequencing of *B. cepacia* strain GG4 was performed recently using Roche 454 GS FLX technology. The assembly of the genomic data produced an approximate genome size of 6.6 Mb with 72 contigs (Hong et al., 2012a). This plasmid-free bacterium was found to consist of two chromosomes with G + C content of 66% and 2,716 predicted coding sequences. The genome sequences corresponding to chromosomes 1 and 2 have been deposited in GenBank, with the accession numbers CP003774 and CP003775, respectively.

The objectives of the present study were to decipher the genomic architecture of strain GG4 for autoinducer protein and subsequently the molecular characterization of this single putative *luxI* homologue, *burI*. The *burI* gene was amplified from genomic DNA of strain GG4 and the gene was overexpressed in *E. coli*. The recombinant BurI protein was purified and the production of AHLs was characterized using mass spectrometry.

## Materials and Methods

### Bacterial strains and culturing conditions

All bacterial strains and plasmids used in this study are listed in Table S1. *B. cepacia* sp. strain GG4 was grown aerobically in Lysogeny Broth (LB) medium or LB agar (Merck, Germany) at 25 °C with shaking (220 rpm). *E. coli* strains were grown routinely in LB medium supplemented with 100 µg/ml ampicillin (Sigma, St. Louis, MO) alone or 30 µg/ml kanamycin (Sigma, St. Louis, MO) and 34 µg/ml chloramphenicol (Sigma, St. Louis, MO), and incubated at 37 °C aerobically with shaking (250 rpm). All bacterial strains were stored frozen at −70 °C in LB supplemented with 50% glycerol.

### Isolation of genomic DNA

An overnight culture of strain GG4 was harvested and lysed with DNAzol reagent (Invitrogen, USA) followed by addition of Proteinase K (NEB, USA). Absolute ethanol was added to the lysate to precipitate the DNA. The resulting DNA pellet was washed twice with 75% (v/v) ethanol, air-dried and dissolved in TE buffer (pH 8.0) and stored at 4 °C. Plasmid DNA for use in subcloning was isolated using QIAprep Spin Miniprep Kit (Qiagen, Germany) according to manufacturer’s instructions. The quality of extracted DNA was analyzed by means of agarose gel electrophoresis, followed by ethidium bromide (Sigma, St. Louis, MO) staining. The purity of the DNA was estimated by NanoDrop spectrophotometer (Thermo Scientific) and the yield was estimated using Qubit 2.0 Fluorometer (Life Technologies, USA).

### Construction of recombinant burI expression plasmids

The *burI* gene was amplified from the extracted genomic DNA of *B. cepacia* GG4 using polymerase chain reaction (PCR). The primers used were *burI*-F (5′ CCATGGGCATGCGGACCTTCGTTCAC3′) and *burI*-R (5′ CTCGAGTATGGCGGCGATGGCTT3′). The primers were designed based on the sequence of *burI* identified from whole genome analysis. Two restriction sites (underlined), NcoI and XhoI, were added to the forward and reverse primers, respectively. The PCR cycles consist of an initial denaturation at 95 °C for 5 min, followed by 30 cycles at 95 °C for 30 s, annealing at 55 °C for 40 s and extension at 72 °C for 40 sec, and a final extension at 72 °C for 5 min. Sterile deionised water was used as the negative control in all PCR reactions. The PCR product was verified using agarose gel electrophoresis followed by ethidium bromide (Sigma, St. Louis, MO) staining. The amplicon with the desired band size was purified using QIAquick Gel Extraction kit (Qiagen, Germany) and ligated to pGEMT (Promega, USA), as per the manufacturer’s instructions. The resulting recombinant plasmid (designated pGEMT-*burI*) was transformed into *E. coli* JM109 (Sambrook & Russel, 2001). A DNA fragment was excised from this recombinant plasmid by digestion with NcoI and XhoI followed by gel purification, and ligated into pET28a (Novagen, Germany) digested with the same enzymes, to produce pET28a-*burI*. Verification for the correct insert cloned into pGEMT and pET28a plasmids was done by automated Sanger DNA sequencing.

### Nucleotide sequence and bioinformatics analysis of *burI*

The nucleotide sequences of *burI* and other *luxI* homologues were verified using the BLASTX program available from NCBI website (http://www.ncbi.nlm.nih.gov/). Searches for open reading frame (ORF) was performed using the ORF Finder tool (http://www.ncbi.nlm.nih.gov/gorf/gorf.html) while the fundamental properties of the proteins were predicted using ExPASy (http://www.expasy.org/). Multiple sequence alignments of the amino acid sequences were performed using Sequence Manipulation Suite (http://www.bioinformatics.org). A phylogenetic tree of the BurI protein was then constructed with Molecular Evolutionary Genetic Analysis (MEGA) version 5.0 using Neighbour-Joining strategy (Chan et al., 2010; Tamura et al., 2011). Bootstrap analyses up to 1,000 replicates were used to provide a good confidence estimates for the constructed tree.

### Heterologous expression of BurI protein in *E.coli*

To express the His-tagged fusion protein, pET28a-*burI* was transformed into *E. coli* BL21 (DE3)pLysS cells (Sambrook & Russel, 2001). Then, 1 ml of the overnight culture was inoculated into fresh 50 ml of LB medium containing both kanamycin and chloramphenicol and cells were grown to an OD_600_ of 0.5. Optimization on overexpression of BurI was performed in terms of isopropyl-D-thiogalactopyranoside (IPTG; Sigma, St. Louis, MO) concentration (0.2–1.0 mM) and temperature of induction (15 °C and 37 °C). Once IPTG was added, growth of the culture was continued for 8 h with shaking at the desired temperature. The cells were harvested by centrifugation at 10,000 rpm and lysed by BugBuster™ Protein Extraction Reagent supplemented with protease inhibitors and Benzonase nuclease (Novagen, Germany). The recombinant proteins were purified from cell lysate using Ni-IDA agarose affinity (Applied Biological Materials Inc., USA) according to manufacturer’s protocol.

### Sodium dodecyl sulfate polyacrylamide gel electrophoresis (SDS- PAGE) analysis

Samples of the cell lysates taken before and after IPTG induction were suspended and boiled in 5× Laemmli sample buffer (Bio-Rad, USA), and examined by polyacrylamide gel electrophoresis (PAGE; Bio-Rad, USA) in the presence of SDS on 12.5% (w/v) gels (Laemmli, 1970). Following this, Coomassie brilliant blue R-250 (CBB; Bio-Rad, USA) was used to stain the gel before viewing.

### AHL extraction

*E. coli* BL21 cells harboring pET28a-*burI* were cultured in LB medium buffered with 50 mM 3-[*N*-morpholino] propanesulfonic acid (MOPS) to pH 6.5 to prevent hydrolysis of AHL in alkaline medium (Yates et al., 2002). The culture was then induced with IPTG as described earlier. The spent culture supernatant was extracted thrice using equal volume of acidified ethyl acetate (0.1% v/v glacial acetic acid; Merck, Germany) and the organic solvent was evaporated to dryness. The dried extracts were then resuspended in 1 mL of acidified LC-ethyl acetate and allowed to dry. Finally, 100 µL of acetonitrile (HPLC grade; Merck, Germany) was added to dissolve the extracted AHLs. The mixture was then filtered with a 0.22 µm syringe filter and an aliquot (20 µL) of the extract was placed in a sample vial for analysis using liquid chromatography mass spectrometry (LC-MS) (How et al., 2015).

### Identification of AHL profile by liquid chromatography mass spectrometry (LC-MS/MS)

An Agilent 1290 Infinity LC system (Agilent Technologies Inc., USA) was used as the LC delivery system coupled with an Agilent ZORBAX Rapid Resolution HT column (2.1 mm × 50 mm, 1.8 µm particle size). The column temperature was maintained at 37 °C and the injection volume was set to 2 µL. The analysis was performed in 15 min at a constant flow rate of 0.3 mL/min. The following mobile phases were used: (A) 0.1% v/v formic acid in HPLC grade water and (B) 0.1% v/v formic acid in acetonitrile (ACN). A gradient profiles with the following settings were applied (time: mobile phase A: mobile phase B: 0 min: 80:20, 7 min: 50:50, 12 min: 20:80, and 14 min: 80:20). The high-resolution electrospray ionization mass spectrometry (ESI-MS) was performed with the Agilent 6490 Triple-Quad LC-MS/MS system (Agilent Technologies Inc., USA). ESI in positive mode was employed and the range of *m*/*z* value for precursor ions was set from 150 to 400. The probe capillary voltage was set at 3 kV, sheath gas at 11 mL/h, nebulizer pressure at 20 psi, and desolvation temperature at 200 °C. The collision energy was optimized in 5 eV and fragmentation was performed at 380 eV. For detection of AHLs, precursor ion scan mode was used. The presence of product ion at *m*/*z* 102 corresponds to the presence of [*M* + *H*]^+^ ion of the core lactone ring moiety. Agilent MassHunter software was used for the MS data analysis by comparison of extracted ion (EI) mass spectra and retention index with data obtained from synthetic AHL compounds (Chong et al., 2012; Yin et al., 2012). The ACN and AHL extracted from culture supernatant of *E. coli* harboring pET28a alone were used as the blank and negative controls, respectively.

## Results

**Figure 1:**
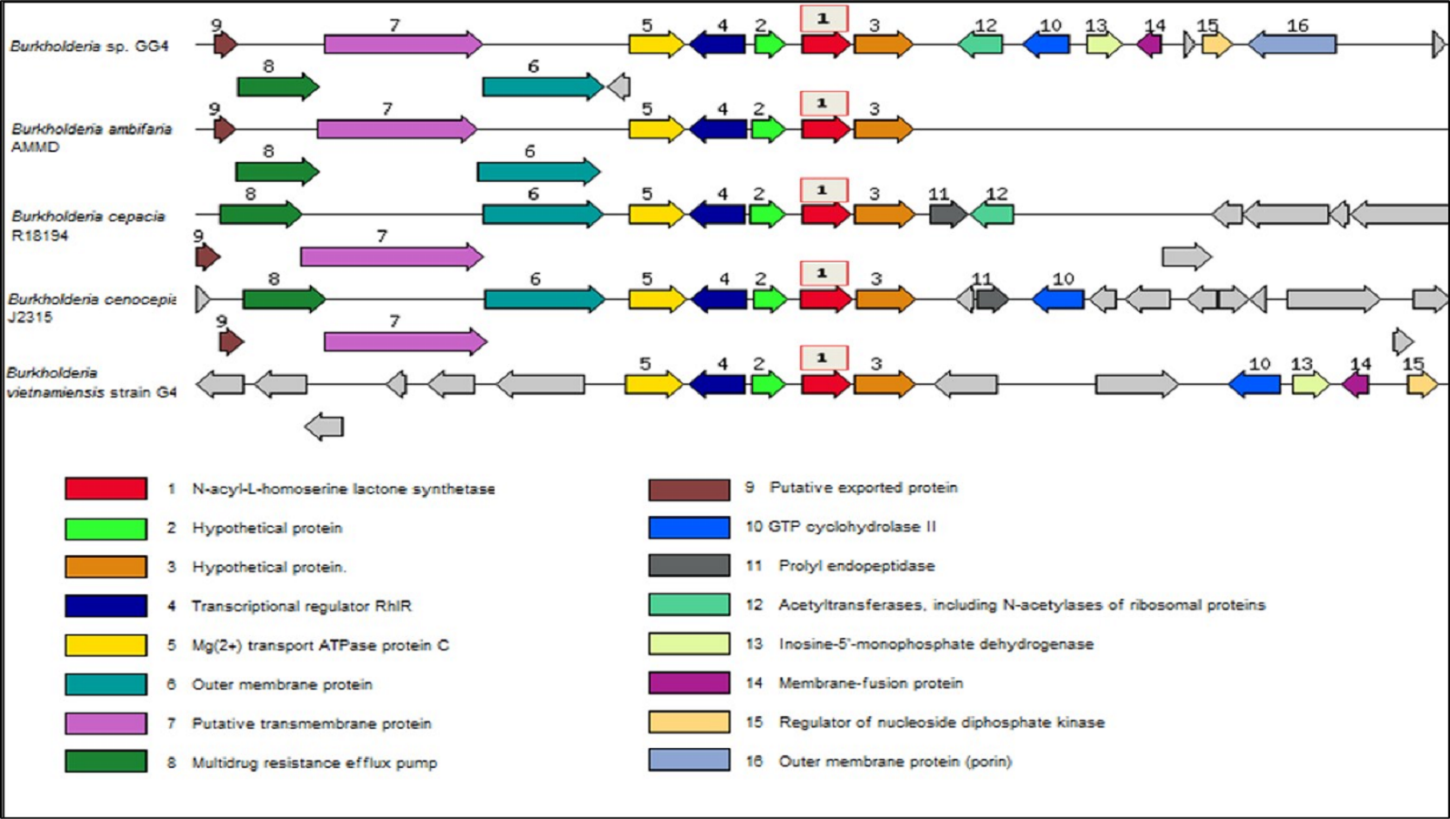
Comparison of LuxI gene cluster among *B. cepacia* GG4 and four close relatives, *B. ambifaria* AMMD, *B. cepacia* R18194,*B. cenocepaci*a J2315 and *B. vietnamiensis* G4. Homologous proteins are shown as the same color. All autoinducer proteins (highlighted boxes), together with LuxR homologue, were found on chromosome 2 of each strain.

From *in-silico* analysis, an open reading frame (ORF) coding for a putative LuxI homologue, designated as *burI*, was found in chromosome 2 of the complete genome sequence of *B. cepacia* strain GG4 (Hong et al., 2012a). This autoinducer synthesis protein has been deposited in the GenBank database (Accession number YP_006617833.1). Analysis of the LuxI gene cluster shows indistinct variation among strain GG4 and other close relatives of *Burkholderia* species (Fig. 1). All the *Burkholderia* strains studied possess *luxI* homologues which are divergently oriented with the upstream transcriptional regulator, *luxR* homologues.

**Figure 2:**
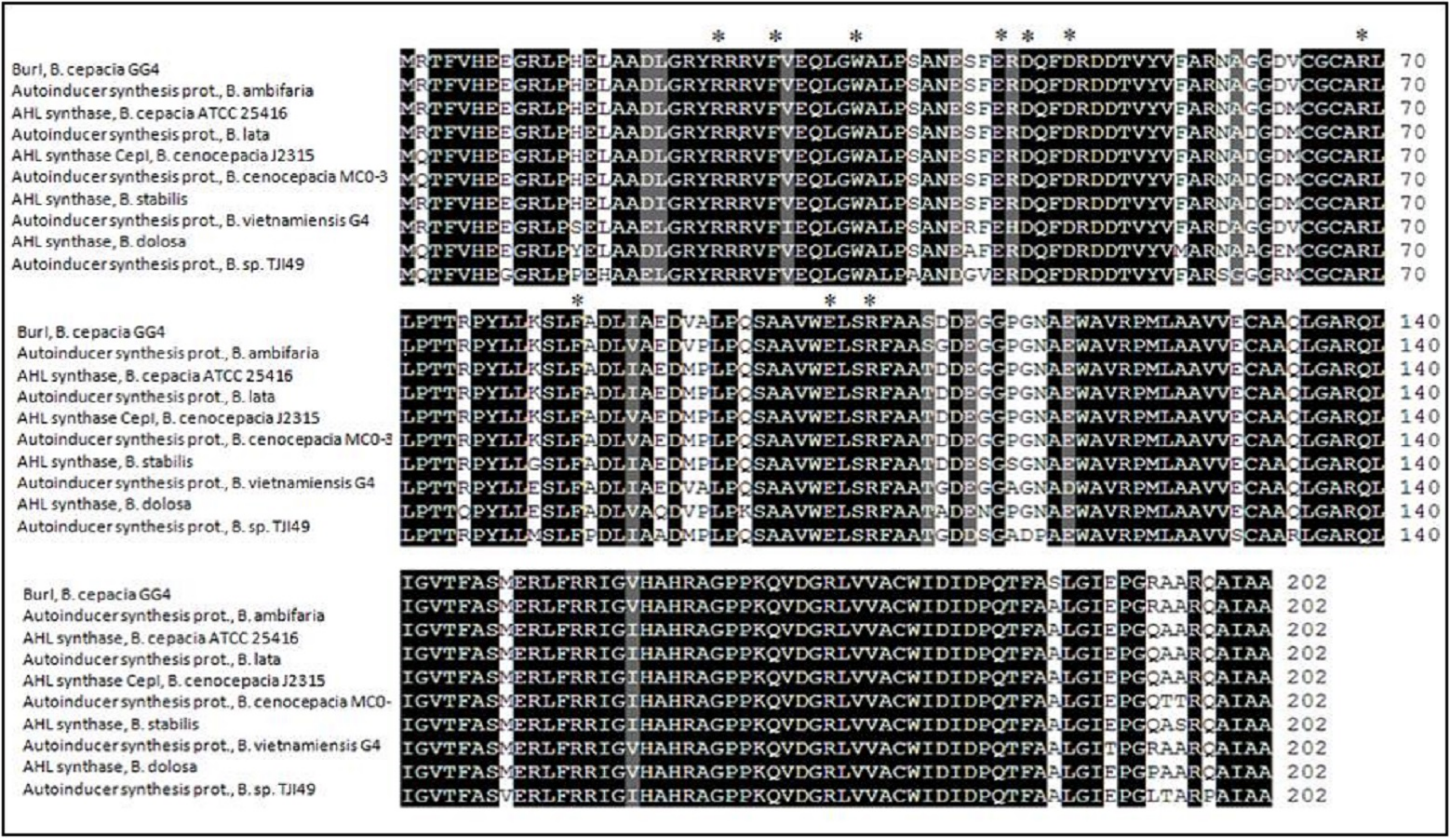
Multiple sequence alignment of *N*-acylhomoserine lactone autoinducer protein sequences of *Bukholderia cepacia* GG4 and other *Bukholderia* species. Sequences were derived from NCBI database (http://www.ncbi.nlm.nih.gov) and were aligned using Sequence Manipulation Suite (http:// www.bioinformatics.org). Identical residues are shaded in black and those that are similar among the sequences are given a gray background. The 10 invariant amino acid residues of LuxI homologs are denoted with asterisks. GenBank accession numbers (in parentheses): Autoinducer synthesis protein from *B. ambifaria* (ABI89671.1), AHL synthase from *B. cepacia* ATCC 25416 (AAG61123.1), autoinducer synthesis protein *B. lata* (YP_371808.1), *N*-acylhomoserine lactone synthase CepI from *B. cenocepacia* J2315 (YP_002234481.1), autoinducer synthesis protein *Burkholderia cenocepacia* MC0-3 (YP_001779189.1), AHL synthase from *B. stabilis* (AAG61126.1), autoinducer synthesis protein *B. vietnamiensis* G4 (YP_001117676.1), acyl-homoserine-lactone synthase *B. dolosa* (WP_006766138.1), autoinducer synthesis protein *Burkholderia* sp. TJI49 (EGC99688.1).

**Figure 3:**
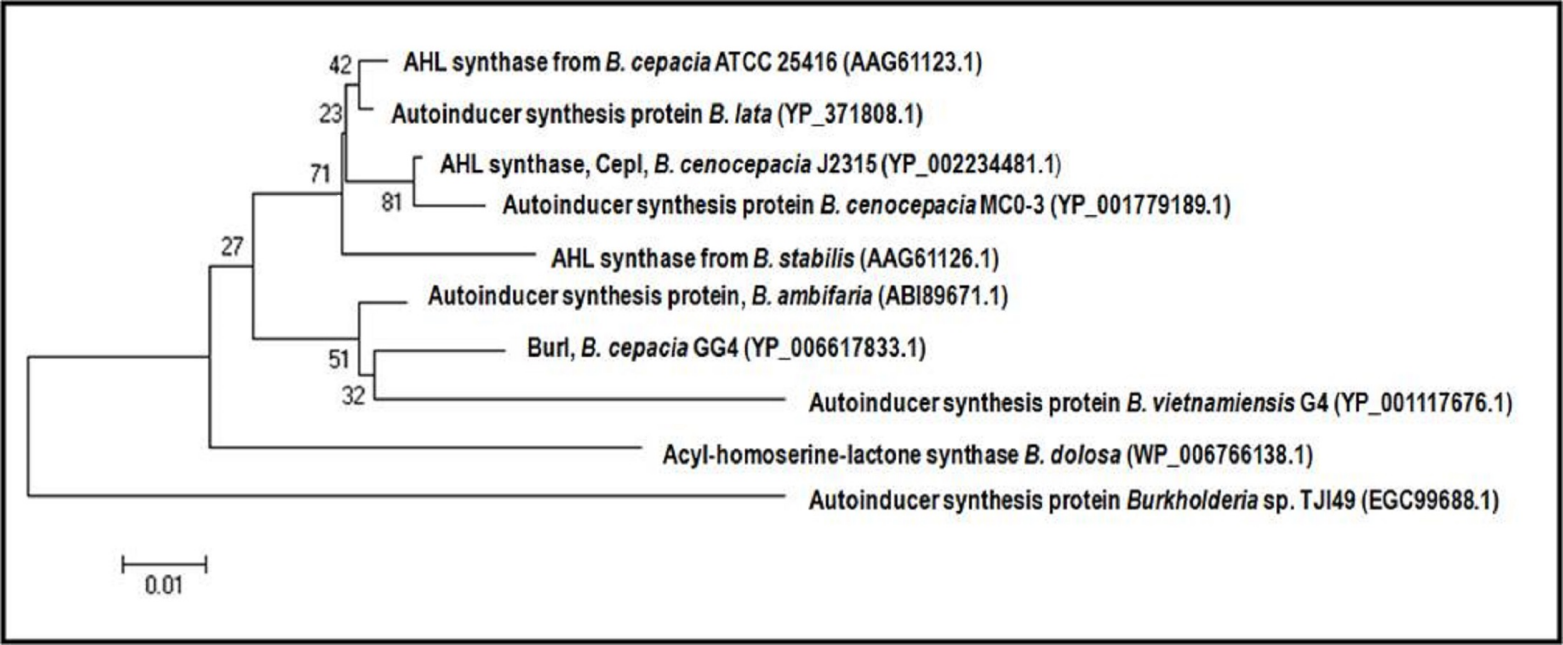
Phylogenetic tree generated using Neighbour-Joining algorithm, showing the phylogenetic position of the putative AHL synthase of *B. cepacia* strain GG4. The tree was drawn to scale, with branch lengths in the same units as those of the evolutionary distances used to infer the phylogenetic tree. The horizontal bar at the bottom represents evolutionary distance as 0.01 change per nucleotide position. The numbers at the nodes indicate the bootstrap values as percentage of 1,000 replications. GenBank accession numbers are indicated in parentheses.

Web-based similarity searches against the GenBank database indicated that BurI protein sequence is highly homologous to other AHL synthase of *Burkholderia* species, mostly from *B. cepacia complex* (Bcc) strains. All Bcc strains have been isolated from both environmental and human clinical sources (Coenye & Vandamme, 2003). Multiple sequence alignments in Fig. 2 illustrated that BurI protein shares similarities and conserved amino acids with other reported AHL synthase of *Burkholderia* species. It was found that BurI and all the LuxI family members contain the conserved 10 amino acid residues of LuxI homologues. On the other hand, the phylogenetic tree constructed based on amino acid alignment (Fig. 3) illustrated that BurI was clustered closely with autoinducer synthesis protein from *B. vietnamiensis* G4, a type of nitrogen-fixing bacteria colonizing the rhizosphere of rice (Suárez-Moreno, Caballero-Mellado & Venturi, 2008).

**Figure 4:**
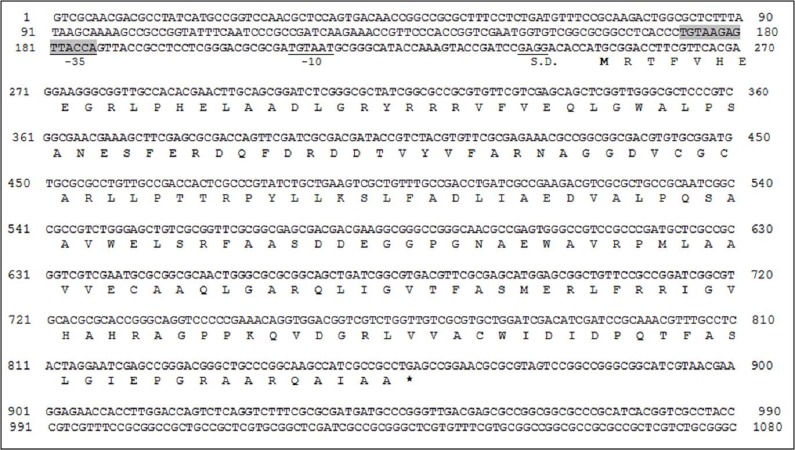
The nucleotide sequence of *Bulkholderia* sp. strain GG4 *burI* gene and its flanking sequence. Single letter codes for deduced amino acid sequence are shown below the nucleotide sequence. The stop codon (TGA) is marked by an asterisk. The translational start site (**M**) is in bold and S.D. denotes the putative Shine-Dalgarno site. The proposed core promoter elements, −10 and −35 boxes are underlined. The 20-bp *lux* box-like sequence in the promoter region is shown is shaded and overlaps the putative −35 region.

Figure 4 shows the upstream and downstream sequences of *burI* gene. The *burI* gene codes for a putative AHL synthase which consists of 202 amino acids. The sequence, TGTAAT, at 34 nucleotides upstream from the start codon and the sequence, TTACCA, located at 65 nucleotides upstream could correspond to the putative −10 and −35 transcriptional sequences, respectively. There are 25 nucleotides separating the two consensus regions, in agreement to the optimum spacing suggested by Hawley & McClure (1983) on *E. coli* promoter analysis. A potential Shine-Dalgarno site (GAGG) is located 6 bp upstream from the start codon. Apart from that, a putative *lux* box (TGTAAGAGTTACCAGTT) was found to partially overlap the putative −35 region. This sequence is identical to the *lux*-box of *B. cepacia* isolated by Lewenza et al. (1999) and matching the consensus *lux* box in 15 of 20 positions.

**Figure 5:**
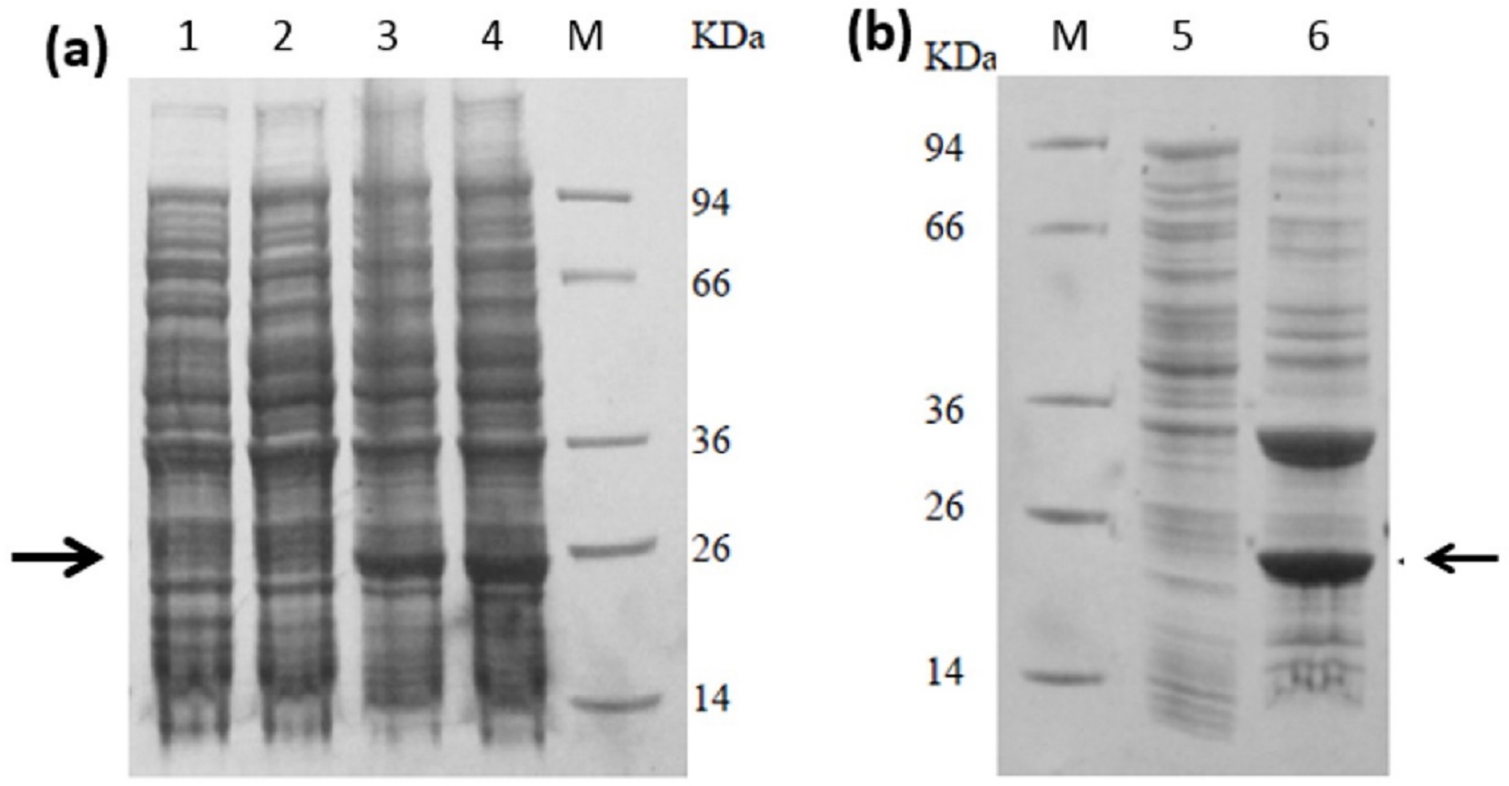
SDS-PAGE profile of overproduction of BurI in *E. coli* BL21 (DE3) pLysS followed by CBB staining. SDS-PAGE analysis on (A) cell lysate and (B) on soluble and insoluble fraction of cell lysate after centrifugation at 14,000 rpm. Cell lysate of *E. coli* harboring pET28a-*burI* without IPTG induction (lane 1); overnight IPTG induction at 15 °C (lane 2); overnight IPTG induction at 37 °C (lanes 3 and 4); soluble fraction of *E. coli* harboring pET28a-*burI* with overnight IPTG induction at 37 °C (lane 5); insoluble fraction of *E. coli* harboring pET28a-*burI* with overnight IPTG induction at 37 °C (lane 6); protein marker in kDa (lane M). The BurI protein was found to be overexpressed at approximately 25 kDa in inclusion bodies.

**Figure 6:**
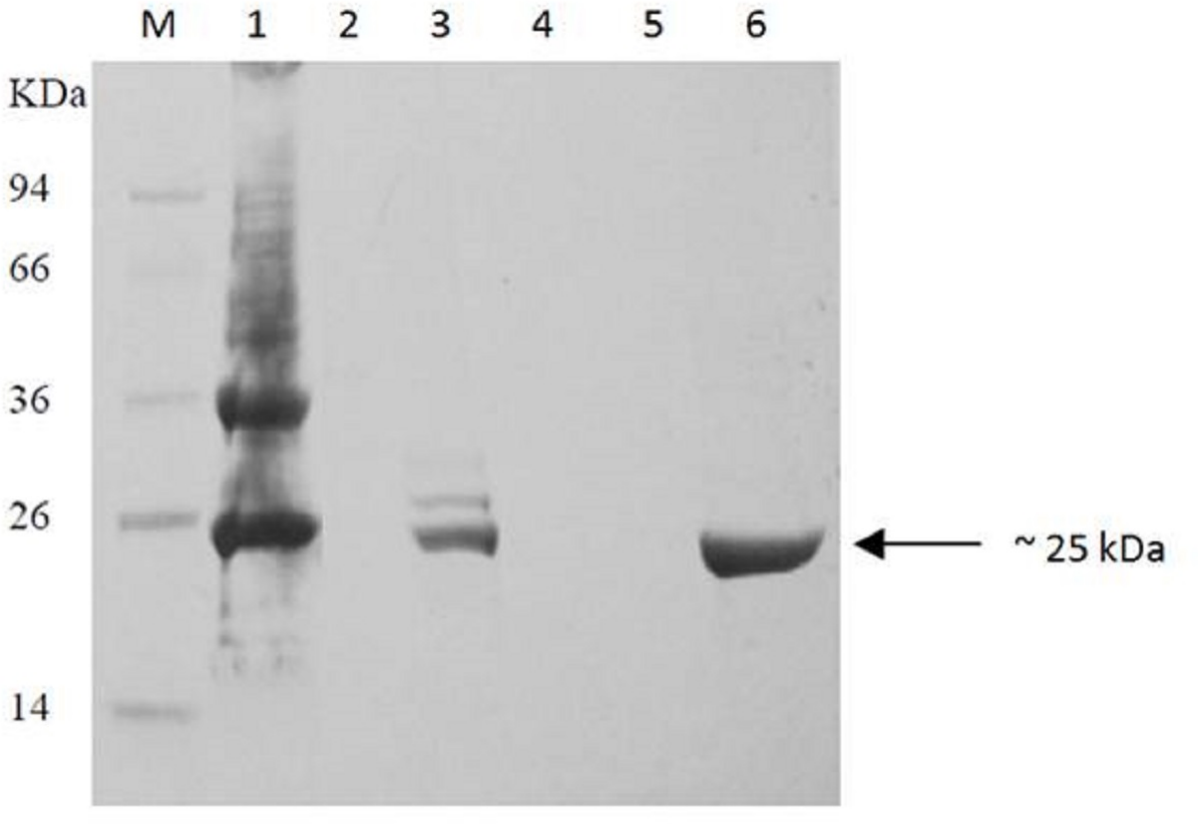
Purification of recombinant BurI protein from insoluble fraction of induced *E. coli* BL21 harboring pET28a-*burI*. Lane M, protein marker in kDa; Lane 1, precipitation dissolved in 8M urea; lane 2, flow through; lane 3, resin after elution step; lane 4, wash fraction using 8M urea, lane 5, wash fraction using 8M urea containing 20 mM imidazole; lane 6, eluted fraction using 8M urea containing 500 mM imidazole. The recombinant BurI protein was successfully purified from its inclusion bodies with fairly good purity.

The 609 bp-ORF of *burI* was then amplified by PCR and cloned into pET28a overexpression vector, producing pET28a-*burI*, with expression of a 6 × His-tag driven by a T7 promoter. This recombinant plasmid was transformed into *E. coli* BL21 and the recombinant *burI* gene was overexpresed upon IPTG induction. The optimum induction was found to be at 1 mM IPTG (data not shown). Figure 5 shows the presence of the overexpressed protein from the harvested cell lysate in the SDS-PAGE, corresponding to the recombinant BurI protein. However, the protein was mostly present in the insoluble fraction. Hence, purification from the precipitation dissolved in 8 M urea was performed using Ni-IDA agarose affinity chromatography (Fig. 6). The purity of the recombinant protein was fairly good with molecular weight approximtely 25 kDa, inclusive of His-tag peptide.

**Figure 7:**
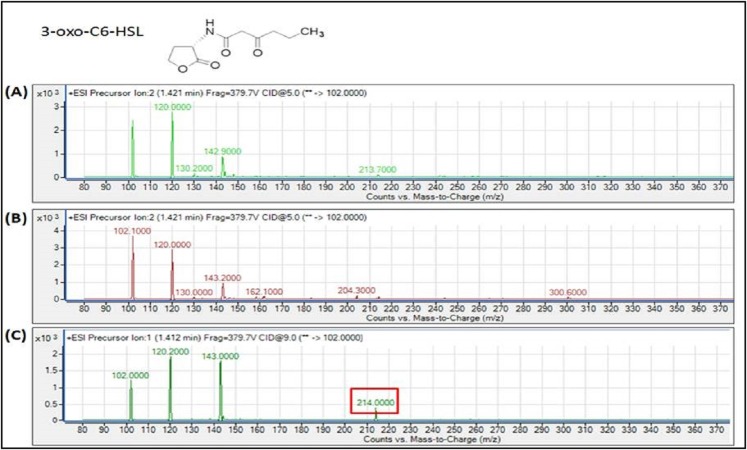
MS analyses of the extract of spent culture supernatant from IPTG-induced *E. coli* BL21 harboring pET28a-*burI*. By comparing with the corresponding synthetic AHL standard, the mass spectra demonstrated the presence of 3-oxo-C6-HSL at *m*/*z* 214.0000. (A) Mass spectra of *E. coli* BL21 harboring pET28a alone (control); (B) mass spectra of non-induced *E. coli* BL21 harboring pET28a-*burI* (control); (C) mass spectra of induced *E. coli* BL21 harboring pET28a-*burI*.

**Figure 8:**
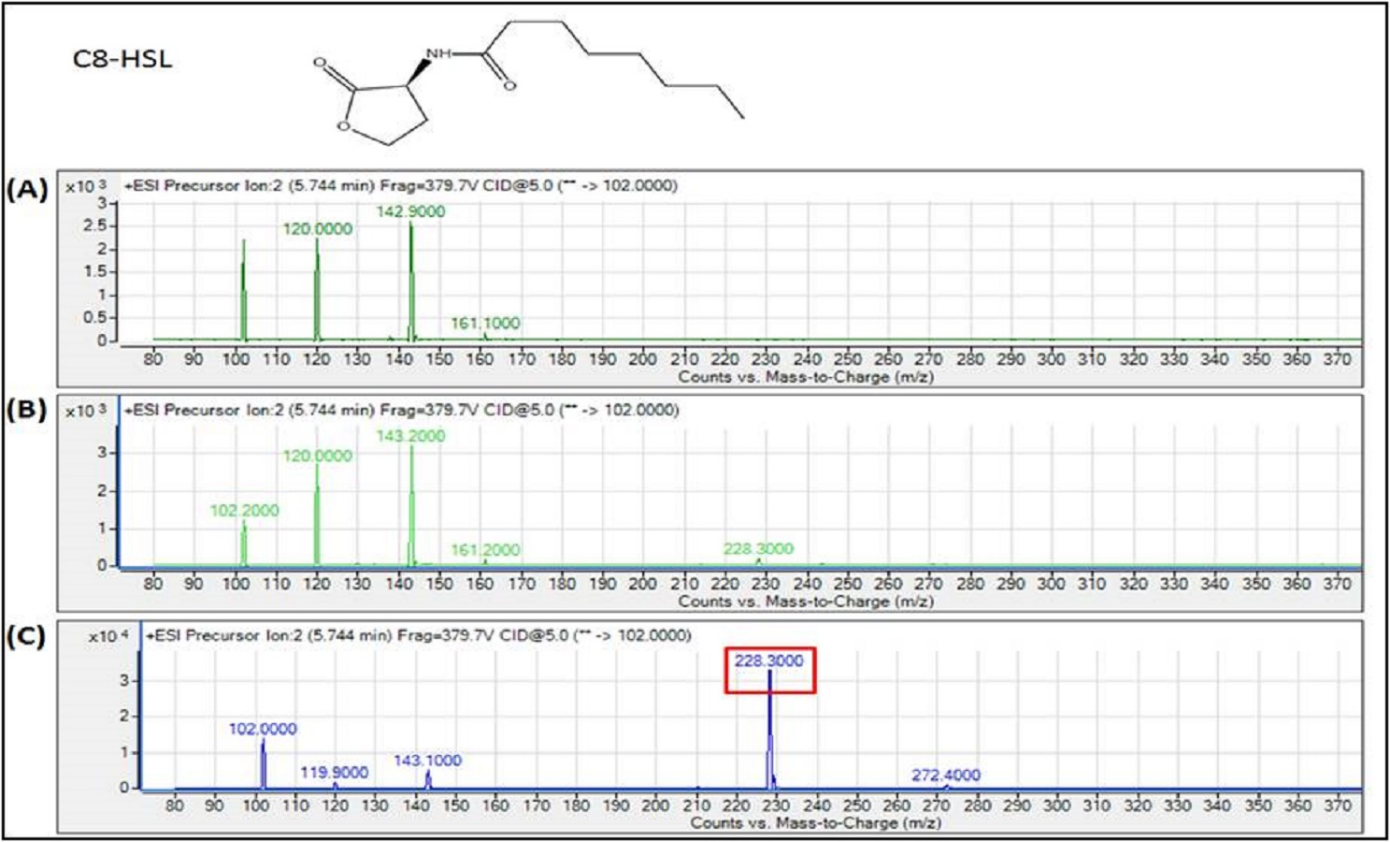
MS analyses of the extract of spent culture supernatant from IPTG-induced *E. coli* BL21 harboring pET28a-*burI*. By comparing with the corresponding synthetic AHL standard, the mass spectra demonstrated the presence of C8-HSL at *m*/*z* 228.3000. (A) Mass spectra of *E. coli* BL21 harboring pET28a alone (control); (B) mass spectra of non-induced *E. coli* BL21 harboring pET28a-*burI* (control); (C) mass spectra of induced *E. coli* BL21 harboring pET28a-*burI*.

**Figure 9:**
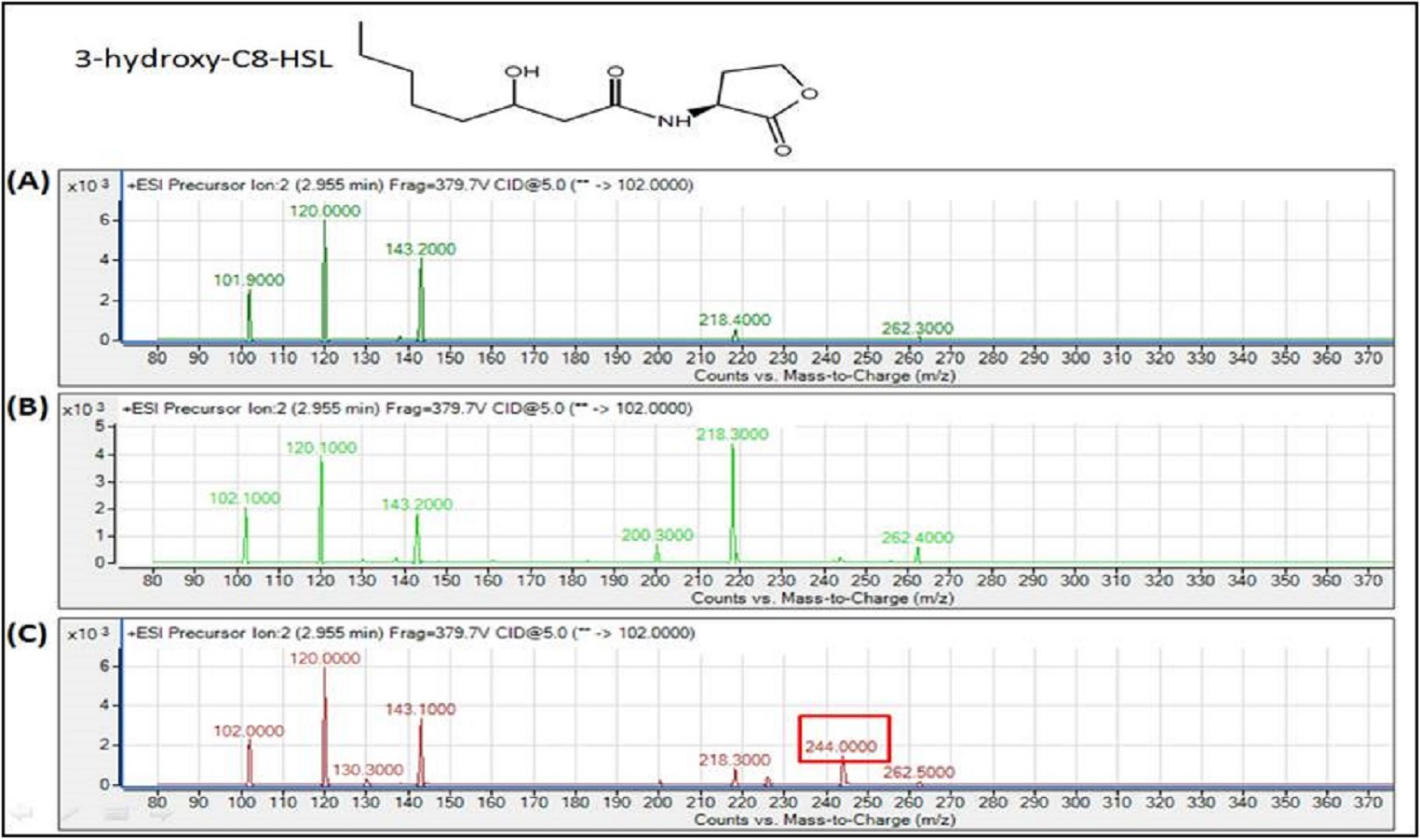
MS analyses of the extract of spent culture supernatant from IPTG-induced *E. coli* BL21 harboring pET28a-*burI*. By comparing with the corresponding synthetic AHL standard, the mass spectra demonstrated the presence of 3-hydroxy-C8-HSL at *m*/*z* 244.0000. (A) Mass spectra of *E. coli* BL21 harboring pET28a alone (control); (B) mass spectra of non-induced *E. coli* BL21 harboring pET28a-*burI* (control); (C) mass spectra of induced *E. coli* BL21 harboring pET28a-*burI*.

The spent culture supernatants of the IPTG-induced *E. coli* BL21 harboring pET28a-*burI* were analyzed using Agilent 6490 Triple-Quad LC-MS/MS system. High resolution mass spectrometry analysis demonstrated the presence of 3-oxo-hexanoyl-homoserine lactone (3-oxo-C6-HSL), *N*-octanoyl-L-homoserine lactone (C8-HSL) and 3-hydroxy-octanoyl-homoserine lactone (3-hydroxy-C8-HSL) with *m*/*z* values of 214.0000, 228.3000 and 244.0000, respectively (Figs. 7–9). The mass spectra of the extracted AHL were similar to the corresponding synthetic compounds at their respective retention times (Fig. S1). For each detected AHL, a fragment ion at *m*/*z* 102 was observed, which correponds to the lactone moiety. AHLs were not found in the *E. coli* BL21 harboring pET28a alone or appeared in trace amounts in noninduced *E. coli* harboring pET28a-*burI*. The mass spectra also revealed quantitatively that C8-HSL was produced more abundantly than the other two AHLs after 8 h of induction. Nevertheless, C9-HSL was found to be absent in the culture supernatant of *E. coli* BL21 harboring pET28a-*burI* despite several optimizations in induction of the protein expression (Fig. S1).

## Discussion

Members of the *Burkholderia* genus of Proteobacteria are nutritionally versatile and they are ubiquitous in the environment. The unusually large genomes, which often consist of several (typically two or three) large replicons, as well as the ability to use different kinds of compounds as energy sources (Parke & Gurian-Sherman, 2001) are believed to be the main reasons of the ecological versatility of these bacteria. The ability to survive in a diverse array of environments is partly attributed to QS activity. QS via AHL signalling molecules is present in almost all *Burkholderia* species. It plays a crucial role in governing virulence and other phenotypic traits such as colonization and niche invasion. This cell-dependent communication enables rapid adaptation of the organisms to different changes in environment (Choudhary et al., 2013). In a recent study, Hartmann & Schikora (2012) demonstrated that QS signals may induce various types of plant responses.

QS-associated genes in *Burkholderia* sp. are located on chromosome 2 where most genes related to virulence and secretion systems are previously found (Whitlock, Mark Estes & Torres, 2007). Thus far, it has been known that there are two major AHL QS systems in the genus *Burkholderia*. The first system is CepI/R system found in members of the Bcc which produce and respond to C8-HSL (Eberl, 2006; Venturi et al., 2004). The other system is the BraI/R system that produces and responds to 3-oxo-C12-HSL found in many diazotrophic and plant-associated *Burkholderia* species (Caballero-Mellado et al., 2004). According to Suarez-Moreno et al. (2010), CepI/R system is a global regulatory system in *Burkholderia* sp. On the other hand, the BraI/R system is stringently regulated by RsaL and this system was believed to control regulation of a small set of genes.

Besides the conserved CepI/R system, additional QS systems were found in other Bcc strains. For instance, Malott & Sokol (2007) reported the presence of BviI/R system in *B. vietnamiensis*, while some *B. cenocepacia* strains harbor the CciIR system (Malott et al., 2005). Apart from that, a number of *B. cenocepacia* strains have been shown to produce two additional types of QS signalling molecules, 2-heptyl-4-quinolone (HHQ) and cis-2-dodecenoic acid (BDSF) (Deng et al., 2009; Diggle et al., 2006). A study has shown that absence of HHQ from *B. pseudomallei* altered its bacterial colony morphology and increased the synthesis of elastase (Diggle et al., 2006). This shows that a single type of QS signal can be secreted in different kinds of bacteria and a bacterial strain in fact could harbor more than one type of signaling system. On the other hand, it is interesting to know that Bcc strains could recognize and respond to *P. aeruginosa* QS molecules, indicating a possible inter-species communication among the etiological agents that contribute to the disease in cystic fibrosis patients (Riedel et al., 2001). Hence, it could not be denied that the QS activity exhibited by strain GG4 in our study is a way of communication with other members of the microbial communities in the soil rhizosphere.

One of the earliest studies reported that three types of AHLs were detected from spent culture supernatant of *B. cepacia*, namely *N*-butyryl-L-homoserine lactone (C4-HSL), 3-oxo-C6-HSL and *N*-hexanoyl-L-homoserine lactone (C6-HSL) (McKenney, Brown & Allison, 1995). However, two studies on *B. cepacia* strain K56-2 using several autoinducer bioassays showed the presence of C8-HSL and C6-HSL (minor product) expressed by CepI/R system (Lewenza et al., 1999; Lewenza & Sokol, 2001). This autoinducer CepI/R system was the first described QS system in the *Burkholderia* genus (Lewenza et al., 1999). It was reported that the production of several extracellular virulence factors, including lipase, protease, and siderophores such as pyochelin, salicylic acid, and ornibactin were under the control of CepI/R system.

In addition, the CepI/R system from *B. cepacia* strain H111, a cystic fibrosis respiratory isolate, has been found to be involved in controlling biofilm formation and swarming motility. Similarly, two AHL molecules, C8-HSL and C6-HSL, were produced by this strain but in a ratio of 10:1. It was also shown that if the CepI mutant was defective in secretion of AHL, biofilm formation was affected significantly (Huber et al., 2001). However, these defects were restored to wild-type phenotype when synthetic C8-HSL was added into the growth medium. On the other hand, the presence of QS system with the secretion of C8-HSL and C6-HSL was reported in onion pathogen *B. cepacia* strain ATCC 25416. The *cep* locus is implicated in protease production and onion pathogenicity via the expression of polygalacturonase, an extracellular enzyme responsible for onion maceration. It was reported that proteolytic activity was significantly lower in CepI mutant and hence, attenuated onion pathogenicity. Likewise, the complemented mutant harboring the *cepI* locus in *trans* had caused a higher rate of polygalacturonase activity and onion maceration (Aguilar, Bertani & Venturi, 2003).

In the current study, *burI* which encodes the putative AHL synthase has been successfully cloned and characterized. The estimated size of the purified protein was in agreement with the SDS-PAGE profile (Fig. 6). The deduced protein sequence has a high degree of homology with several AHL synthases from other Bcc strains. This strongly indicates a conserved QS system and low rate of random mutation for this autoinducer gene among the heterogeneous Bcc. It appears likely that these proteobacteria share similar basic QS mechanism and gene regulation in AHL synthesis although they are responsible for different target genes. Analysis of the completed genome sequences revealed that BurI is probably the only member of the LuxI family (Hong et al., 2012a). The genetic organization of GG4 and other *Burkholderia* species shows clearly that majority of the *luxI/R* gene clusters are conserved (Fig. 1).

A detailed analysis of both upstream and downstream sequences of *burI* gene identified a putative Shine-Dalgarno region as well as −10 and −35 promoter elements (Fig. 4). Although both −10 and −35 promoter regions are not strongly conserved, the sequences meet the requirement of the typical *E. coli* RNA polymerase *σ*^70^ consensus promoter sequences (Harley & Reynolds, 1987). The palindromic sequence of *lux* box upstream of the gene may highly suggest that the putative transcriptional activator, BurR binds to *burI* promoter to activate *burI* expression. Such hypothesis, although yet to be validated, is in agreement with findings from CepI/R system in *B. cepacia* (Lewenza et al., 1999), in which the expression of *cepI* is activated by CepR/AHL complex. In fact, in many Bcc members, it is found that transcription of *luxI* homologue which is activated by LuxR/AHL complex provides a signal amplification via a positive feedback mechanism (Choudhary et al., 2013). The increase in the production of AHL is important in response to cell density and expression of target genes. Lewenza and co-workers (1999) postulated that the expression of pvdA, a gene involved in the biosynthesis of ornibactin in *B. cepacia*, was regulated by CepR as the promoter region of pvdA****contains a possible *lux* ****box-like sequence. Meanwhile, protease activity was also found to be influenced by CepR as mutation in *cepR* result in a protease-negative phenotype. Hence, the regulation of ornibactin biosynthesis and protease activity by QS system in strain GG4 opens another research scope for future study.

While most *Burkholderia* strains synthesize C6-HSL and C8-HSL (Suarez-Moreno et al., 2010), GG4 strain produces other AHL such as C9-HSL. Such behavior in many *Burkholderia* species highly suggests that the AHL synthase is not well-conserved. When *E. coli* harboring the recombinant *burI* was induced with IPTG for 8 h and its spent supernatants was assayed with LC-MS/MS, the presence of 3-oxo-C6-HSL, C8-HSL and 3-hydroxy-C8-HSL were confirmed, suggesting the BurI is indeed the AHL synthase of *B. cepacia* strain GG4. Such findings are in consistent with a recent study by Chan et al. (2011a) which obtained the same AHL profile. Nevertheless, in this work, C9-HSL which was secereted by the parent strain could not be detected from the spent culture supernatant of *E. coli* harboring the recombinant *burI.* Most likely, this discrepancy can be attributed to gene expression in different species used in this study. It is possible that *E. coli* produces low amounts of C9-HSL or that the growing conditions employed were not optimal for synthesis of this autoinducer molecule. Another likely reason is that *E. coli* cells may not have the biosynthetic machinery needed to activate the production of C9-HSL. A point noteworthy is C8-HSL appeared to be the AHL synthesized in highest amount by *E. coli* harboring *burI*, which was in agreement with numerous studies that most Bcc isolates produce C8-HSL in greatest abundance.

Phylogenetic analysis (Fig. 3) demonstrated that BurI is closely associated to autoinducer protein of *B. vietnamiensis* G4, an environmental isolate which play important roles in nitrogen fixation (Malott & Sokol, 2007). In addition to C8-HSL and C6-HSL, *B. vietnamiensis* G4 strain produce long chain AHL molecules such as *N*-decanoyl-homoserine lactone (C10-HSL), *N*-dodecanoyl-homoserine lactone (C12-HSL), and 3-oxo-decanoyl-homoserine (3-oxo-C10-HSL) (Malott & Sokol, 2007). It is interesting to find that strain GG4 is the only soil isolate among Bcc strains to synthesize C9-HSL. Such differences in AHL profile is believed to express a different QS network which regulates diverse physiological processes and to facilitate intercellular communication among bacterial communities in the rhizosphere environment.

Chan et al. (2011a) reported that quorum-quenching was found to co-exist with AHL-dependent QS in *B. cepacia* strain GG4. This isolate was able to reduce 3-oxo-AHLs to the corresponding 3-hydroxy compounds. From LC-MS/MS analysis, this study demonstrated that the production of 3-hydroxy-C8-HSL was directed by the LuxI homologue and not by the reduction of 3-oxo-C8-HSL. As there could be strain variation in terms of AHL production by *B. cepacia*, it would be of great interest to look into the relationship between different strains of Bcc isolates and their autoinducer synthesis.

To date, characterization of Bcc species in the environment has been more limited than investigation on clinical epidemiology. A study by Stoyanova et al. (2007) reported that grasses and maize from the Gramineae group are essential rhizospheric hosts and niche for Bcc bacteria. Our group, hence, believes that environmental isolates such as strain GG4 are likely to have a major impact on the properties of polymicrobial communities in the rhizosphere. In fact, many Bcc isolates have been exploited for various purposes, including plant growth promotion, biological control of plant pathogens, and bioremediation of recalcitrant xenobiotics (Stoyanova et al., 2007). In the future, it is plausible that more Bcc strains associated with different host plants will be isolated from different habitats.

Despite significant progresses on the taxonomy of Bcc, the knowledge of the virulence determinants and their molecular mechanisms used by Bcc bacteria, particularly the clinical strains, remains scarce. Hence, as *B. cepacia* is ubiquitous in nature, it is an attractive organism to study the role of QS as the global regulatory system in controlling virulence, thereby developing the interventions designed to combat infection or to induce beneficial applications in agriculture.

## Supplemental Information

10.7717/peerj.1117/supp-1 Figure S1 MS analyses of C9-HSL on the extract of spent culture supernatant from IPTG-induced *E. coli* BL21 harboring pET28a-*burI* By comparing with the corresponding synthetic AHL standard at *m*/*z* 244.0000, the mass spectra demonstrated the absence of C9-HSL from culture supernatant of *E. coli* BL21 harboring pET28a-*burI*. (a) Mass spectra of *E. coli* BL21 harboring pET28a alone (control); (b) mass spectra of non-induced *E. coli* BL21 harboring pET28a-*burI* (control); (c) mass spectra of induced *E. coli* BL21 harboring pET28a-*burI*. C9-HSL was not detected from culture supernatant of *E. coli* BL21 harboring pET28a-*burI*

10.7717/peerj.1117/supp-2 Figure S2 Chromatogram and mass spectra showing the peaks of the synthetic AHLs (3-oxo-C6-HSL, 3-hydroxy-C8-HSL and C8-HSL (marked by arrows)) (A) Chromatogram of the synthetic standards used in this study. Peaks corresponding to 3-oxo-C6-HSL, 3-hydroxy-C8-HSL and C8-HSL were shown at their respective retention times. (B) mass spectrum of 3-oxo-C6-HSL showing *m*/*z* 214.2000. (C) mass spectrum of C8-HSL showing *m*/*z* 228.2000. (D) mass spectrum of 3-hydroxy-C8-HSL showing *m*/*z* 244.1000.

10.7717/peerj.1117/supp-3 Table S1 Bacterial strains and plasmids used in this study

## Additional Information and Declarations

### DNA Deposition

The following information was supplied regarding the deposition of DNA sequences: Genbank, accession no. CP003774 and CP003775.
