# Supplementary material for: Whole genome sequencing enables the characterization of BurI, a LuxI homologue of Burkholderia cepacia strain GG4
Source: PeerJ. 2015 Aug 6;3:e1117. doi: 10.7717/peerj.1117 (PMC4540015; doi:10.7717/peerj.1117)
Supplement: Figure S1 — By comparing with the corresponding synthetic AHL standard at m/z 244.0000, the mass spectra demonstrated the absence of C9-HSL from culture supernatant of E. coli BL21 harboring pET28a-burI. (a) Mass spectra of E. coli BL21 harboring pET28a alone (control); (b) mass spectra of non-induced E. coli BL21 harboring pET28a-burI (control); (c) mass spectra of induced E. coli BL21 harboring pET28a-burI. C9-HSL was not detected from culture supernatant of E. coli BL21 harboring pET28a-burI [file peerj-03-1117-s001.pdf]

Whole genome sequencing enables the characterization of *burI, a LuxI* homologue of *Burkholderia cepacia* strain GG4

Kah Yan How*, Kar Wai Hong and Kok-Gan Chan

Correspondence:
Kah Yan How,
Email: hkyan@um.edu.my, Tel. +603-79675162; Fax: +603-79674509

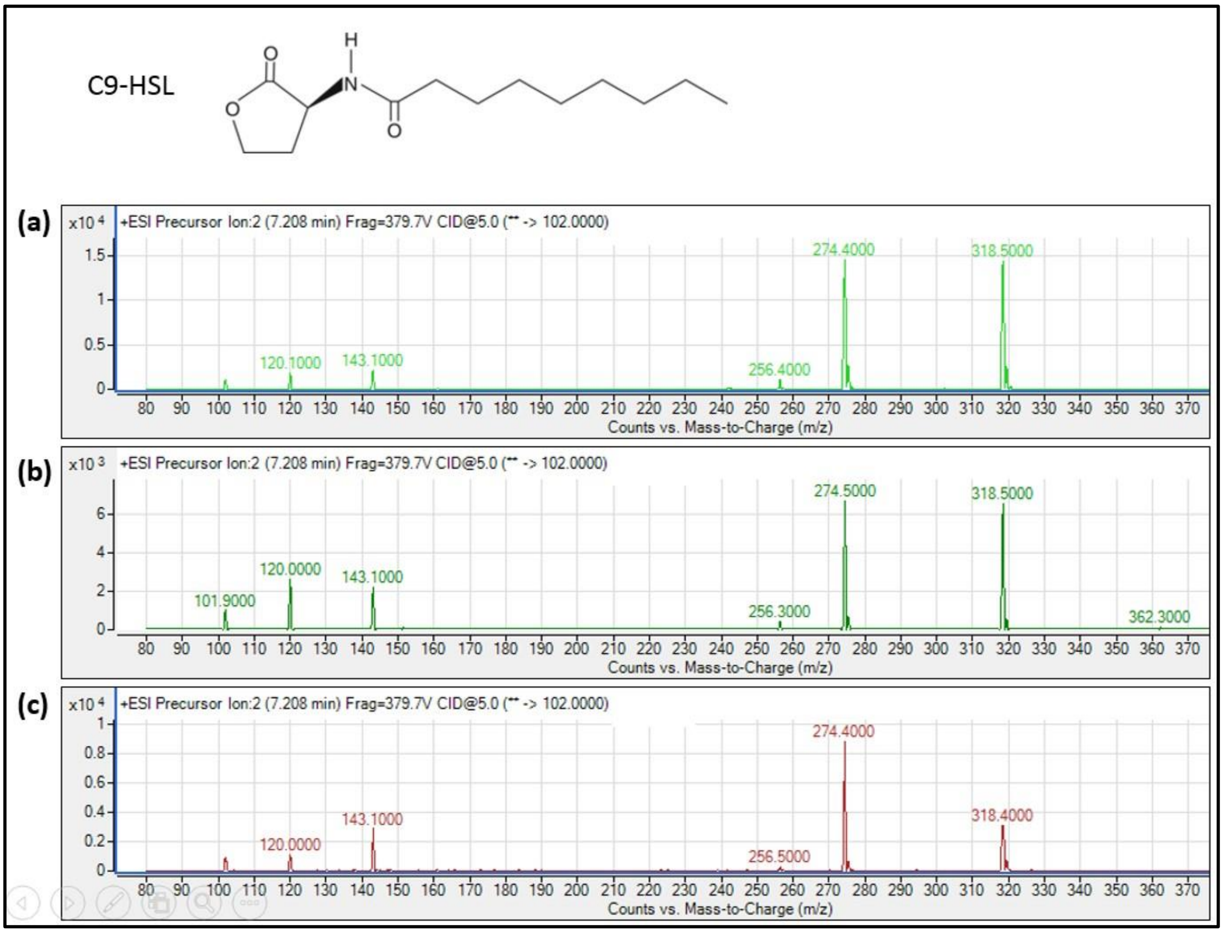

Figure S1: MS analyses of C9-HSL on the extract of spent culture supernatant from IPTG-induced *E. coli* BL21 harboring pET28a-burI. By comparing with the corresponding synthetic AHL standard at *m/z* 244.0000, the mass spectra demonstrated the absence of C9-HSL from culture supernatant of *E. coli* BL21 harboring pET28a-burI. (a) Mass spectra of *E. coli* BL21 harboring pET28a alone (control); (b) mass spectra of non-induced *E. coli* BL21 harboring pET28a-burI (control); (c) mass spectra of induced *E. coli* BL21 harboring pET28a-burI. C9-HSL was not detected from culture supernatant of *E. coli* BL21 harboring pET28a-burI
