# Supplementary material for: Whole genome sequencing enables the characterization of BurI, a LuxI homologue of Burkholderia cepacia strain GG4
Source: PeerJ. 2015 Aug 6;3:e1117. doi: 10.7717/peerj.1117 (PMC4540015; doi:10.7717/peerj.1117)
Supplement: Figure S2 — (A) Chromatogram of the synthetic standards used in this study. Peaks corresponding to 3-oxo-C6-HSL, 3-hydroxy-C8-HSL and C8-HSL were shown at their respective retention times. (B) mass spectrum of 3-oxo-C6-HSL showing m/z 214.2000. (C) mass spectrum of C8-HSL showing m/z 228.2000. (D) mass spectrum of 3-hydroxy-C8-HSL showing m/z 244.1000. [file peerj-03-1117-s002.pdf]

Supplementary Figure S2

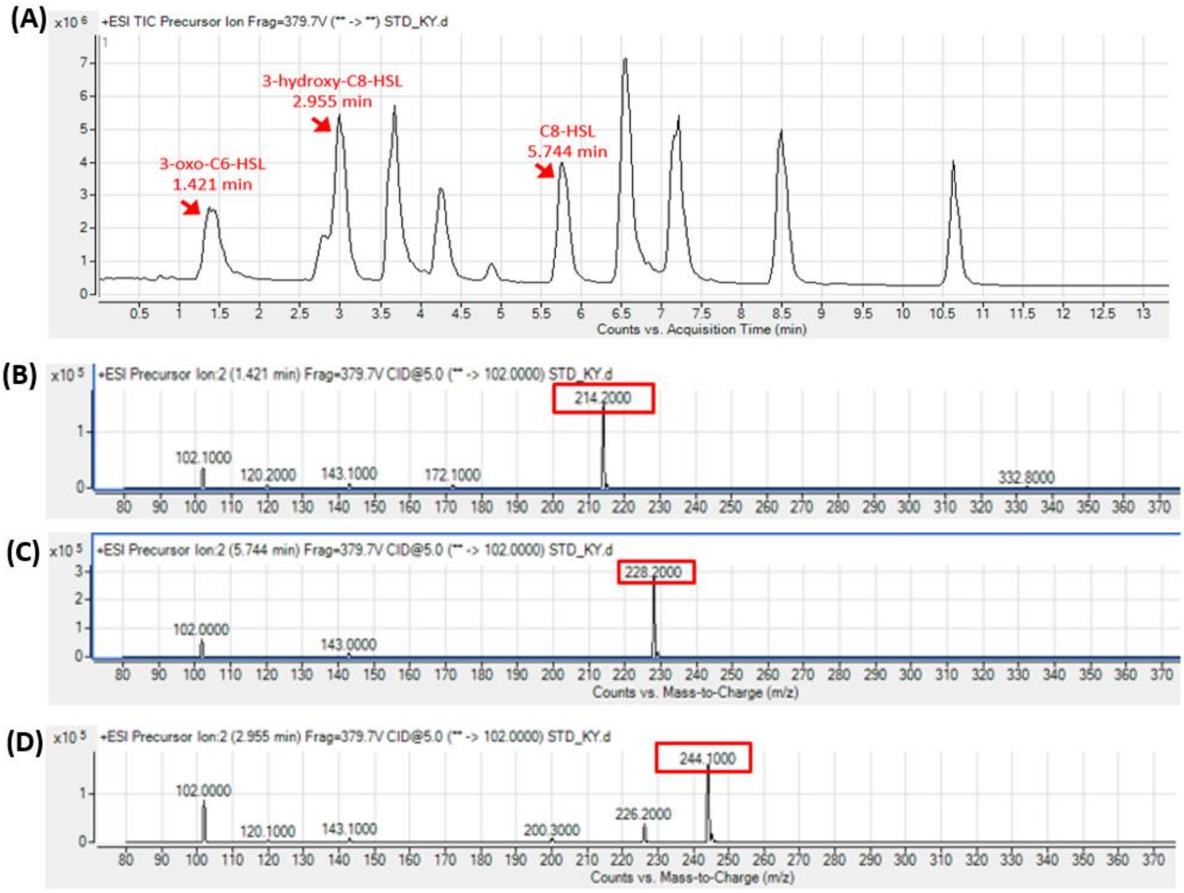

Figure S2. Chromatogram and mass spectra showing the peaks of the synthetic AHLs (3-oxo-C6-HSL, 3-hydroxy-C8-HSL and C8-HSL, marked by arrows). (A) Chromatogram of the synthetic standards used in this study. Peaks corresponding to 3-oxo-C6-HSL, 3-hydroxy-C8-HSL and C8-HSL were shown at their respective retention times). (B) mass spectrum of 3-oxo-C6-HSL showing *m/z* 214.2000. (C) mass spectrum of C8-HSL showing *m/z* 228.2000. (D) mass spectrum of 3-hydroxy-C8-HSL showing *m/z* 244.1000.
