# Supplementary material for: Whole genome sequencing enables the characterization of BurI, a LuxI homologue of Burkholderia cepacia strain GG4
Source: PeerJ. 2015 Aug 6;3:e1117. doi: 10.7717/peerj.1117 (PMC4540015; doi:10.7717/peerj.1117)
Supplement: Table S1 [file peerj-03-1117-s003.docx]

**Table S1. Bacterial strains and plasmids used in this study**

| Strain/plasmid | Genotype/description | Source/reference |
| --- | --- | --- |
| *B. cepacia GG4* | Soil isolate | Chan *et al.* (2011) |
| ***E. coli*** |  |  |
| JM109 | endA1 glnV44 thi-1 relA1 gyrA96 recA1 mcrB^+^ Δ(lac-proAB) e14- [F' traD36 proAB^+^ lacI^q^ lacZΔM15] hsdR17(r_K_^-^m_K_^+^) | NEB (USA) |
| BL21 (DE3)pLysS | F^-^ ompT gal dcm lon hsdS_B_(r_B_^-^ m_B_^-^) λ(DE3) pLysS; Cm^R^ | Novagen (Germany) |
| **Plasmids** |  |  |
| pGEMT | TA cloning vector, Amp^r^ | Promega |
| pGEMT-*burI* | pGEMT containing 651 bp of *burI* with NcoI-BamHI sites | This study |
| pET28a | Circle plasmid carrying N-terminal His-tag/ thrombin/enterokinase configuration plus an optional C-terminal His tag sequence; Km^r^ | Novagen (Germany) |
| pET28a-*burI* | pET28a containing 651 bp of *burI* cloned into NcoI-BamHI sites | This study |

^a^Amp^r^, Cm^r^, and Km^r^ indicate resistance to ampicillin, chloramphenicol, and kanamycin, respectively
